# A scoring system for euthanasia decisions in rescued raccoon dogs (*Nyctereutes procyonoides*): Standardized criteria in wildlife rehabilitation

**DOI:** 10.1371/journal.pone.0349230

**Published:** 2026-05-28

**Authors:** Bong Kyun Kim, Soo Hyung Eo

**Affiliations:** 1 Department of Forest Science, Kongju National University, Yesan, Chuncheongnam-do, Korea; 2 Department of Companion and Laboratory Animal Science, Kongju National University, Yesan, Chuncheongnam-do, Korea; 3 Chungnam Wild Animal Rescue Center, Yesan, Chuncheongnam-do, Korea; University of Lincoln - Brayford Campus: University of Lincoln, UNITED KINGDOM OF GREAT BRITAIN AND NORTHERN IRELAND

## Abstract

Wild animal rescue and control centers routinely encounter situations that require evaluating a rescued animal’s potential for recovery and making decisions on appropriate treatment, rehabilitation, or euthanasia. However, currently, scientific standards to guide these decisions are lacking, and frequent reliance on empirical judgment leads to application of subjective criteria for euthanasia. Therefore, this study aimed to analyze factors affecting rescue outcomes in 1,376 raccoon dogs rescued by the Chungnam Wild Animal Rescue Center over 13 years, to predict the likelihood of release, and to develop a system to inform euthanasia decisions. The model that best predicted the release of rescued raccoon dogs included the cause of the accident, season, and level of injury. Based on this, a scored assessment support system was constructed, and raccoon dogs were classified into a ‘low-risk group,’ requiring active treatment and rehabilitation owing to the high likelihood of live release, a ‘medium-risk group,’ requiring mid-to-long-term euthanasia decisions through close monitoring and re-assessment, and a ‘high-risk’ group, for which immediate euthanasia may be considered because of the extremely low probability of release. The proposed scoring-based euthanasia decision support system integrates clinical and situational factors and remains practical by relying on data that can be readily obtained in the field immediately after rescue. Therefore, by supporting rapid, consistent decision-making and using basic data to prepare standardized euthanasia guidelines, this system can improve animal welfare and operational efficiency.

## Introduction

Wild animal rescue and rehabilitation are important conservation activities that facilitate the recovery of individual animals from accidents or injuries before their release back into natural habitats [1–3]. However, in cases where the likelihood of recovery is minimal, where severe disability or sequelae are anticipated, when treatment and rehabilitation may result in excessive distress, or when exposed to disaster situations such as large-scale wildfires that substantially increase the risk of mortality, euthanasia may be a necessary intervention [4–6]. Rapid, logical decisions regarding euthanasia for rescued wild animals are important for animal welfare because they prevent excessive or unnecessary suffering and positively contribute to the efficient utilization of limited resources, in terms of both finances and personnel [7]. For similar reasons, the International Wildlife Rehabilitation Council (IWRC) and the National Wildlife Rehabilitators Association (NWRA) include the right to euthanasia in their ethical codes, enabling animal welfare to be maintained and preventing distress in wild animals incapable of returning to nature [8]. Therefore, euthanasia decisions require careful consideration of both the ethical perspective related to animal welfare and the practical issue of efficient resource management, making them a key challenge for improving animal welfare and fulfilling ethical responsibilities [9,10]. To develop effective euthanasia criteria for wild animals, it is essential to initially determine the conditions under which the likelihood of death considerably exceeds the potential for successful rehabilitation and release. However, actively applying relevant discussions or studies in the field is challenging, and euthanasia decisions are frequently based on relatively simple clinical criteria, such as the presence or absence of severe disability or sequelae, including the loss of two or more legs, permanent loss of sight, or defects in a large number of permanent teeth [4]. Although the level of injury or disability is naturally a critical consideration, an overreliance on veterinary diagnostic outcomes can become a limitation. In some instances, the suitability of euthanasia can be conclusively determined only after treatment has been initiated.

Raccoon dogs (*Nyctereutes procyonoides* Gray, 1834), members of the family Canidae, are predominantly distributed in East Asia, but their range has now extended to Europe, where they are known as an invasive species [11,12]. Canids regulate ecosystem balance as mesopredators [13], are important indicator species of environmental change as they respond sensitively to habitat fragmentation and climate change [14], and are characterized by their varied interactions with humans [15]. Considering these characteristics, and because raccoon dogs are the only wild canid species currently living in South Korea, they require active protection and management. However, following water deer (*Hydropotes inermis* Swinhoe, 1870), raccoon dogs are the second most frequently rescued wild mammals in South Korea [16]. Approximately 60% of rescued raccoon dogs do not recover and die or are euthanized [17]. The high rescue frequency and mortality rates indicate the need to review the suitability of treatment and rehabilitation protocols for rescued raccoon dogs and develop methods to predict their chances of release. To minimize unnecessary distress in rescued animals, the criteria for euthanasia decisions must be established based on scientific evidence. Previous studies analyzing factors affecting rescue outcomes in raccoon dogs have identified injury level and accident cause as key determinants of release. Individual characteristics such as sex or age, as well as seasonal and environmental conditions, have also been suggested as influential variables [17]. These studies provide considerable academic value by systematically delineating the various factors affecting the release of rescued raccoon dogs. However, their scope has been primarily theoretical, lacking quantitative methods to predict the likelihood of release at the rescue site or to develop scientific criteria for euthanasia decisions.

In this study, rescue outcomes were analyzed across a range of conditions using data collected from dog raccoons rescued by the Chungnam Wild Animal Rescue Center over 13 years (from 2012 to 2024) to develop a scoring system that facilitates informed decision-making regarding euthanasia. Specifically, this study aimed to test the hypothesis that ‘injury level,’ which is generally the most universally considered factor in euthanasia decisions in the field, is the best predictor of raccoon dog release or death. Multiple models, including variables beyond injury level, were constructed to identify the optimal model that most accurately predicts the likelihood of rescue outcomes for raccoon dogs. Additionally, by constructing a scored euthanasia decision support system based on the optimal model, this study aimed to provide scientific evidence to quantitatively predict the likelihood of the release of rescued raccoon dogs and to support decision-making, including euthanasia decisions, in the field.

## Materials and methods

### Conditions affecting rescue outcomes

The animals used in this study consisted of 1,537 raccoon dogs rescued in Chungcheongnam-do and nearby regions between January 2012 and December 2024. After excluding animals that died at the scene, multiple variables, including level of injury, cause of accident, season, and environment, were recorded for the remaining 1,376 animals. These four variables were selected based on their importance and frequency in previous studies that analyzed raccoon dog accidents [17]. Rescue outcomes, such as the likelihood of release, were then analyzed (Table 1). A key distinction between the present study and previous research in terms of data utilization is the inclusion of 200 raccoon dogs rescued in 2024 (108 released and 92 deceased, including euthanasia), whereas previous analyses were limited to data from 2012–2023. These cases account for approximately 15% of the total dataset and constitute a substantial expansion of the available evidence.

**Table 1:** Description of the variables used in the analysis of wild mammals admitted to Chungnam Wild Animal Rescue Center.

Variable	Variable type	Description
Triage	Categorical	Level 1 (Normal), Level 2 (Mild injury), Level 3 (Severe injury), Level 4 (Very severe injury)
Causes of distress	Categorical	7 distinct causes of distress and an ‘Others’ group (comprising 14 additional causes)
Season	Categorical	Spring (March–May), Summer (June–August), Autumn (September–November), Winter (December–February)
Environment	Categorical	Agricultural area, By buildings, Forest, Road, Water bodies, Others
Outcome	Categorical	Dependent variable: release or dead

During the study design phase, several candidate variables were initially considered in addition to the four variables ultimately included in the final analysis. These candidate variables comprised sex, age, time to rescue, distance from rescue location, and body weight. To evaluate their relevance, each variable was subjected to preliminary screening analyses assessing its association with outcome measures and its contribution to model performance. Variables that did not demonstrate statistically meaningful associations or that failed to improve predictive performance were excluded from the final analytical framework.

### Rescue outcomes prediction and preparation of criteria for euthanasia decisions

The raccoon dog rescue outcomes were defined as a binary dependent variable: ‘Release (0)’ or ‘Dead (1).’ Release was defined as a successful return to nature following treatment and rehabilitation. All other outcomes were classified as ‘Dead,’ which included death in care (DIC), death on arrival (DOA), and euthanasia. Specifically, DIC referred to animals who died during treatment and rehabilitation; DOA to animals who died within 24 hours of rescue; and euthanasia to animals who were humanely euthanized owing to disease diagnosis, viral infection, or other untreatable conditions.

This study aimed to develop a model with high explanatory power to predict rescue outcomes and support decision-making regarding treatment or euthanasia, using data collected from rescued raccoon dogs. Accordingly, several candidate models were constructed, and their goodness-of-fit was evaluated. Before the full analysis, we assessed each predictor’s explanatory power for rescue outcomes by fitting single-variable models. Among these, the model including the triage variable demonstrated the highest explanatory power. This variable was therefore selected as the baseline predictor, and additional variables were subsequently incorporated to evaluate their incremental contribution to model performance and to identify the optimal model. Previous studies have also reported this factor as the most influential determinant of rescue outcomes [17], supporting its use as the baseline predictor in the present modeling framework. Therefore, the M1 model included only the single variable ‘Triage.’ Subsequently, the models were sequentially extended by adding variables such as the cause of the accident, season, and environment. M8 was the complete model that included all the variables. Using this approach, eight models, ranging from the univariate model to the complete model, were constructed and compared. The fit of each model was evaluated using Akaike’s Information Criterion (AIC) [18,19], and the model with the lowest AIC value was selected as the optimal model. In addition, ΔAIC and Akaike weights (wi) were calculated to assess the relative support for each model.

To investigate conditions with a much higher likelihood of death than release and to prepare scientific evidence for euthanasia judgments, a custom scoring system for the effects on survival was developed to present the relative risk. In this study, the Acute Patient Physiological and Laboratory Evaluation (APPLE) score method was proposed to quantify the clinical severity in pet dogs hospitalized at a veterinary hospital [20]. The APPLE score was constructed by fitting a multivariate logistic regression model to clinical and physiological indices collected during the first 24 h after admission, selecting statistically significant predictors, and assigning integer scores by scaling the estimated regression coefficients with constant values [20]. This scoring process is a systematic evaluation method that can be used to establish euthanasia judgments and treatment protocols in clinical settings for dogs and cats [21–23]. In this study, the scores were calculated to indicate the relative risk of each condition included in the optimal model. To define the scores, the estimated regression coefficients for each condition were divided by a scaling factor and rounded to the nearest integer. The reference variable (intercept) with the lowest mortality was set to 0, and the other conditions were assigned increasing scores based on their risk, relative to the reference variable [20]. For example, an injury level of ‘Level 1,’ which is the lowest injury level and defined as practically normal, was assigned a score of 0; then, for the more severe injury levels (Levels 2–4), higher scores were assigned in proportion to the increase in the regression coefficient. Consequently, a higher score indicates a higher risk of death. Finally, the scores for all conditions were summed to calculate the ‘custom score.’ A logistic regression model was fitted with the custom score as the independent variable and release as the dependent variable, and mortality (*P*) was estimated [20]. Using the intercept (*β*₀) and slope (*β*₁) estimated in this process, a linear predictor (log odds, *R*) was calculated and entered into the logistic function to obtain a probability. The logistic equation is as follows:


$$\begin{array}{c} P\;=\frac{\text{exp }(R)}{\text{1 }+\text{ exp }(R)},\text{ log odds }(R)\;=\text{ log}(\frac{P}{\text{1 }-\;P})=\;\beta_{\text{0}}\;+\beta_{\text{1}}\;\times \text{ custom score} \end{array}$$

Finally, based on the relationship between mortality and custom scores, a release prediction interval (cutoff) was determined using receiver operating characteristic (ROC) analysis. In the ROC analysis, sensitivity and specificity were the key criteria. Sensitivity is the ratio of actual deaths correctly classified by a prediction model. Specificity is the ratio of actual release cases accurately classified as release using the prediction model. In this study, the cutoff with sensitivity ≥90% was defined as the upper bound for the high-release probability group, and the cutoff with specificity ≥90% was defined as the lower bound for the high-mortality risk group. Therefore, all cases were classified into three categories of ‘low risk,’ ‘medium risk,’ and ‘high risk,’ and the predictions were compared with actual mortality for each category to test the validity of the prediction model. Additionally, the Youden index was used to calculate the optimal cutoff value, and the consistency of the analysis was evaluated.

The assessment support system developed in this manner enables intuitive prediction of the likelihood of death based on the individual animal’s total score and can serve as a practical index for triage and euthanasia decisions. All statistical analyses were performed using R software (version 4.3.3).

This study involved vertebrate animals; however, no live animals were directly handled for research purposes, and no experimental procedures were performed. All data were derived from existing management records of animals rescued and treated at a Wildlife Rescue and Management Center operating in accordance with the relevant laws of the Republic of Korea. As this study constituted a retrospective analysis of such records and did not involve any direct animal experimentation, approval from an institutional animal ethics committee was not required.

## Results

Of the 1,376 total rescued raccoon dogs, 47.3% (651) recovered and were released, while 52.7% (725) eventually died. To identify the model that best explained these rescue outcomes, eight candidate models were constructed, and the AIC values were compared. In this analysis, the M5 model (ΔAIC = 0.0; *w*_*i*_ = 0.766), which included injury level, cause of accident, and season, showed the strongest explanatory power and was selected as the optimal model (Table 2). The M8 model (ΔAIC = 2.38; *w*_*i*_ = 0.233), which included all variables, was also considered an alternative model, whereas the other models showed very low relative support (wi < 0.001), indicating they were not a good fit. The optimal model (M5) was approximately 6.7 × 10^16 times (=0.766/1.1 × 10^-17) more strongly supported than the univariate model with only injury level (M1). This demonstrates that the model, which included additional factors such as the cause of the accident and season, showed considerably superior performance in explaining rescue outcomes compared to the model relying only on the injury level, which is currently the primary variable used in rescue settings. In the ROC analysis of the optimal model, the AUC was 0.763 (95% CI: 0.739–0.788), indicating good discriminative ability. In the Hosmer–Lemeshow test for goodness-of-fit (*χ²* = 8.285, *df* = 6, *p* = 0.218), no statistically significant difference was observed between the predicted and observed values, indicating excellent fit (Table 3). Therefore, the scoring model based on injury level, cause of accident, and season was shown to be valid for predicting the likelihood of release for rescued raccoon dogs.

**Table 2:** Model selection for predicting outcomes of rescued raccoon dogs using logistic regression based on Akaike’s Information Criterion (AIC).

Model	Formula	AIC	ΔAIC	Weight
M5 (Best model)	Triage + Cause + Season	1,593	0.0	0.766
M8 (Full model)	Triage + Cause + Season + Environment	1,595	2.4	0.233
M2	Triage + Cause	1,607	13.8	0.001
M6	Triage + Cause + Environment	1,609	16.5	2.02 x 10^−4^
M7	Triage + Season + Environment	1,633	40.0	1.57 ⅹ 10^−9^
M4	Triage + Environment	1,649	55.9	5.53 ⅹ 10^–13^
M3	Triage + Season	1,656	62.9	1.72 ⅹ 10^–14^
M1	Triage	1,670	77.5	1.12 ⅹ 10^–17^

* The best model showed an AUC of 0.763 and a Hosmer–Lemeshow test result of *χ²* = 8.285, *df* = 6, *p* = 0.218.

**Table 3:** Logistic regression estimates and weighted scores for predictors of mortality based on the best model selected through AIC analysis.

Category	Variable	Estimate	Std. Error	P	score
**Triage**	*Level 1 (Normal)	–	–	–	0
	Level 2 (Mild injury)	0.153	0.331	0.643	2
	Level 3 (Severe injury)	0.895	0.344	0.009	13
	Level 4 (Very severe injury)	1.768	0.335	< 0.001	26
**Causes of distress**	*Isolation in artificial structures	–	–	–	0
	Illegal capture	0.970	0.512	0.058	14
	Orphaning/Kidnapping	1.406	0.475	0.002	20
	Others	1.552	0.492	0.002	22
	Parasitic infection	1.756	0.431	< 0.001	25
	Collision with vehicles	2.916	0.484	< 0.001	42
	Attacks by dogs	4.070	0.884	< 0.001	59
**Season**	*Summer	–	–	–	0
	Autumn	0.589	0.174	0.001	9
	Winter	0.652	0.196	0.001	9
	Spring	0.716	0.179	< 0.001	10

Variables marked with * represent intercept categories used for comparison.

When risk scores were assigned for the factors included in the optimal model, Level 2 showed no significant difference (estimate = 0.153, SE = 0.331, *p* = 0.643) relative to ‘Injury Level 1,’ which was assigned a score of 0 points; however, Level, 3 which was assigned 13 points (estimate = 0.895, SE = 0.344, *p* < 0.001), and Level 4, which was assigned 26 points (estimate = 1.768, SE = 0.335, *p* < 0.001), showed gradually increasing risk. For the cause of the accident, using ‘isolation in artificial structures,’ which had the lowest mortality, as the reference, the risk increased by 14 points for ‘illegal capture’ (estimate = 0.970, SE = 0.512, *p* = 0.06), which was statistically borderline. Meanwhile, scores increased by 20 points for ‘orphaning or kidnapping’ (estimate = 1.406, SE = 0.475, *p* < 0.01), 22 points for ‘other’ (estimate = 1.552, SE = 0.492, *p* < 0.01), and 25 points for ‘parasitic infection’ (estimate = 1.756, SE = 0.431, *p* < 0.001), showing gradually increasing risk. In particular, ‘collision with vehicles’ (estimate = 2.916, SE = 0.484, *p* < 0.001) and ‘attacks by dogs’ (estimate = 4.070, SE = 0.884, *p* < 0.001) were the most fatal conditions for release, associated with scores of 42 and 59 points, respectively. Compared with summer, which had the lowest risk and was assigned a score of 0, fall (estimate = 0.589, SE = 0.174, *p* < 0.001) and winter (estimate = 0.652, SE = 0.196, *p* < 0.01) showed a 9-point increase in risk, while spring (estimate = 0.716, SE = 0.179, *p* < 0.001) showed a 10-point increase in risk. Using the custom score calculated for each animal, the logistic regression equation used to calculate mortality was as follows:


$$\begin{array}{c} R\;=\;-\;3.1505\;+\;(0.0681\;\times \;custom\;score) \end{array}$$

**Fig 1:**
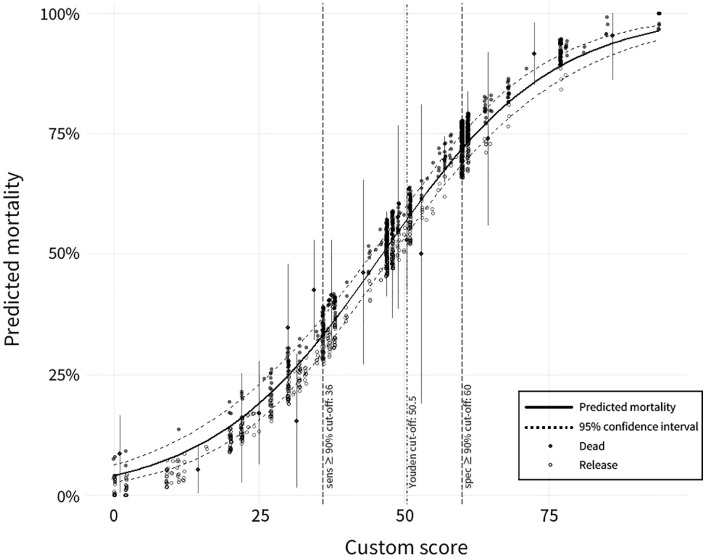
Predicted mortality in rescued raccoon dogs as a function of custom scores, with 95% confidence intervals. Vertical dashed lines indicate cutoffs derived from ROC analysis.

The log odds (*R*) intercept and slope were estimated to be –3.1505 and 0.0681, respectively. The odds ratio was calculated from the slope as follows: exp(0.0681) = 1.07. This indicates that the odds of death for a given animal increased by an average of approximately 1.07-fold for each 1-point increase in the custom score. The mortality (*P*) calculated from the regression equation increased from approximately 4.1% at a custom score of 0 points to 7.8% at 10 points, 14.3% at 20 points, 39.5% at 40 points, and 56.4% at 50 points, showing a gradual S-shaped increase in the predicted mortality with increasing custom scores (Fig 1). Live-release cases were predominantly distributed in the low-score interval, whereas deaths were concentrated in the high-score interval. When the live-release and death-prediction estimates from the optimal model were compared with the observed values, 949 of 1,376 animals (69.0%) showed agreement. When the mortality for each custom score band was analyzed using the cutoff values for this model, the optimal cutoff was 50.5 points, with sensitivity and specificity of 0.68 and 0.71, respectively. Based on this cutoff value, the mortality risk was categorized into three bands. The low-risk band (≤35 points) had a mean mortality rate of 15.4% and showed good consistency between the predicted and observed values (accuracy, 84.6%). The medium-risk band (36–59 points) had a mean mortality rate of 50.3% and an accuracy of 53.6%. The high-risk band (≥60 points) had a mean mortality rate of 75.0% and an accuracy rate of 75.0% (Table 4).

**Table 4:** Mortality probability and decision guidance according to the score band.

N	Score band	Mean mortality	Accuracy	Interpretation
299	≤ 35	15.4%	84.6%	Low risk → treatment/rehabilitation
521	36–59	50.3%	53.6%	Moderate risk → monitoring and re-evaluation
556	≥ 60	75.0%	75.0%	High risk → consider euthanasia

## Discussion

Of the 1,376 rescued raccoon dogs, 47.3% (651 cases) were released after recovery, and 52.7% (725 cases) eventually died. In previous studies on wild mammals [17,24], mortality after rescue was reported to be approximately 65–88%, indicating that the mortality of raccoon dogs in this study was relatively low. This suggests that raccoon dogs exhibit a comparatively higher likelihood of recovery following rescue than other mammalian species, underscoring the importance of active rescue and management for conservation.

The present study was initiated with the hypothesis that injury level, regarded as the most prevalent variable in euthanasia decisions in the field, would be the primary factor that best explains release among rescued raccoon dogs. To test this hypothesis, multiple models were compared, and the model that included injury level, cause of accident, and season showed the strongest explanatory power compared to the model using only injury level. This finding suggests that when predicting the likelihood of release for rescued raccoon dogs, contextual factors, such as the situation at the time of rescue, should be considered, rather than limiting the assessment to veterinary clinical indices alone. In particular, actively collecting clinical indices from rescued wild animals [25], including various field-observed conditions, is challenging. This data collection is essential for objectively understanding each animal’s health status and situation. In summary, the multivariate approach improves the accuracy of rescue outcome predictions and enables precise decision-making in the field.

The scoring system developed to evaluate the likelihood of release for rescued raccoon dogs provided robust classification criteria for predicting an animal’s prognosis based on risk. Among the factors included in the optimal model, the cause of the accident had the highest relative risk. In particular, attacks by dogs, collisions with vehicles, and parasitic infections had the highest scores for all conditions, indicating that these conditions substantially increase the risk of mortality. This is consistent with previous studies indicating that vehicle collisions and parasitic infections are key situational factors affecting raccoon dogs [17]. Therefore, the cause of an accident can be used to predict the prognosis of rescued animals and is a key variable that must be considered in euthanasia decisions. Scores indicating injury-level risk based on the defined cutoffs were also calculated. The scores increased with increasing injury severity, suggesting that appropriate clinical evaluation of the animal at the time of rescue is considerably important for predicting prognosis. The season scores were lower than those for the cause of the accident and the injury level. This indicates that the relative risk associated with the season is reduced. However, apparent seasonal differences were observed; therefore, the season should be considered as a complementary factor alongside the cause of the accident and injury level to improve the explanatory power of release predictions. Raccoon dogs rescued in summer exhibited the highest likelihood of release, whereas animals rescued in the other seasons showed relatively higher mortality rates. Most of the raccoon dogs rescued during the summer were orphaned juveniles who had lost their parents, and cases rescued for this reason had a relatively high release probability. In contrast, during the other seasons, causes associated with higher mortality, such as parasitic infection and vehicle collisions, occurred more frequently. These findings suggest substantial differences in the primary causes of incidents across seasons.

**Fig 2:**
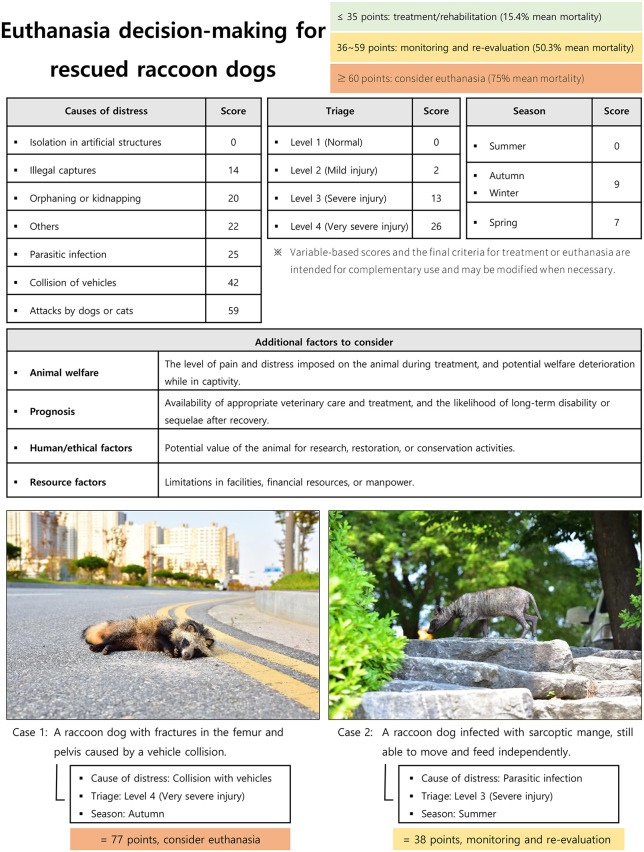
Decision-making framework for euthanasia of rescued raccoon dogs. All images in this figure were created by the authors and are published under the Creative Commons Attribution License (CC BY 4.0).

When the model and scoring system were constructed to predict raccoon dog release, the agreement between the predicted outcomes based on the custom score and the observed outcomes was 69%. This is similar to the 68.6% agreement reported in a previous study that applied the APPLE scores to pet dogs hospitalized at a veterinary hospital [22]. However, in a study of pet dogs undergoing high-flow nasal cannula oxygen therapy, a large discrepancy was observed between the predicted mortality (26%) and the observed mortality (65%) [26]. This suggests that the agreement rate observed in this study was relatively high. Collectively, these findings indicate that the model developed in this study demonstrated robust validity as a prognostic tool, based on a scoring system derived from information obtained during the initial diagnostic evaluation of rescued animals. Thus, considering the relationship between custom scores and mortality, the assessment criteria for each interval were proposed as follows: 1) low-risk group: suitable for active treatment and rehabilitation based on the high likelihood of release, 2) medium-risk group: requires focused monitoring owing to the equal likelihood of either release or death and should be reevaluated based on treatment progression, and 3) high-risk group: euthanasia should be considered before starting treatment because of the high likelihood of death. For example, when a raccoon dog suffered severe injuries following a collision with a vehicle during the fall, the score was calculated to be 77 points, and the predicted mortality rate was approximately 90% (Fig 2). Prompt euthanasia of this animal could be considered a more logical choice than continuing with uncertain treatment.

Collecting diverse clinical and physiological data is notably more limited for wild animals than for pets [25], and when invasive procedures are used to obtain these data, there is an increased risk of capture myopathy, which can negatively affect survival [27]. Therefore, as in the method proposed in the present study, it is practical to include field-observable characteristics or situations when assessing wild animals. This approach is valuable because it integrates both clinical evaluation and situational factors, enabling more objective predictions of the likelihood of release based on scientific evidence. Presenting multidimensional, systematic criteria to supplement the decision-making process, which was previously skewed toward injury level, can also be considered ethically valuable. However, these scores should not be regarded as absolute criteria. In this study, some animals eventually recovered and were released despite having very high customs scores. Therefore, this scoring system should be used as a complementary instrument to support clinical judgment in rescue scenarios.

The method and results of this study provide valuable reference materials for the future development of standardized euthanasia guidelines and criteria for wild animals. However, caution should be exercised regarding overgeneralization. Individual organizations responsible for wild animal rescue and management can use different data collection methods, logging systems, and classification and processing criteria [2,28]. This can lead to differences in outcomes even if the criteria are defined using the same method. Therefore, establishing standardized criteria by having independent organizations conduct their analyses simultaneously, then comparing and consolidating their results, is necessary. This approach would enable wild animal rescue and management centers to collect scientific evidence in key areas of animal care, providing a critical foundation for the establishment of consistent criteria. This study is expected to raise awareness of the need for ethical management strategies that address the welfare of wild animals and to increase academic and practical discussions and efforts to implement them.

## Conclusions

This study presented a scoring system for evaluating scientific evidence and introduced a methodology for predicting the prognosis of rescued raccoon dogs, providing guidance for euthanasia decisions for animals considered unlikely to recover. Contrary to the initial hypothesis that the injury level would be the best predictor, a model that also included the cause of the accident and season exhibited the strongest explanatory power for rescue outcomes among raccoon dogs. The scoring system developed from this model can be used to classify rescued animals into three risk groups based on mortality and predict rescue outcomes with high accuracy. This suggests that this scoring system can provide more objective, quantitative evidence to support euthanasia decision-making processes, which previously relied solely on empirical judgment and clinical assessment. These findings may serve as a foundation for further studies on the applicability of this system to other species beyond raccoon dogs and provide essential data for the development of standardized euthanasia guidelines required for wild animal rescue and rehabilitation.
